# Endosymbiotic bacteria in insects: guardians of the immune system?

**DOI:** 10.3389/fphys.2013.00046

**Published:** 2013-03-15

**Authors:** Ioannis Eleftherianos, Jaishri Atri, Julia Accetta, Julio C. Castillo

**Affiliations:** Insect Infection and Immunity Lab, Department of Biological Sciences, Columbian College of Arts and Sciences, Institute for Biomedical Sciences, The George Washington UniversityWashington, DC, USA

**Keywords:** insect, infection, immunity, endosymbiont, *Wolbachia*, *Spiroplasma*

## Abstract

Insects have evolved obligate, mutualistic interactions with bacteria without further transmission to other eukaryotic organisms. Such long-term obligate partnerships between insects and bacteria have a profound effect on various physiological functions of the host. Here we provide an overview of the effects of endosymbiotic bacteria on the insect immune system as well as on the immune response of insects to pathogenic infections. Potential mechanisms through which endosymbionts can affect the ability of their host to resist an infection are discussed in the light of recent findings. We finally point out unresolved questions for future research and speculate how the current knowledge can be employed to design and implement measures for the effective control of agricultural insect pests and vectors of diseases.

## Introduction

Insects comprise about 95% of all known animal species and are considered one of the most successful groups of living organisms on earth. They possess an extremely efficient immune system that allows them to deal with pathogenic infections. The insect immune system consists of a wide variety of defense mechanisms that act individually or in combination to prevent foreign organisms from entering the insect body or to suppress the growth and replication of pathogens once they gain access to host tissues.

The first line of defense is represented by the insect's epithelia, which serves as a barrier against biotic and abiotic factors, and produce local antimicrobial peptides (AMP) upon infection or wounding (Davis and Engström, 2012). The second line of defense is represented by the innate immune system that responds through a series of mechanisms. These include the systemic production of AMP mainly from the fat body (the insect equivalent to the mammalian liver) as the result of the transcriptional regulation of signaling pathways that are activated upon immune challenge (Ganesan et al., 2011); cellular responses by insect hemocytes (equivalent to mammalian white blood cells) that take part in immune surveillance, phagocytosis, and encapsulation of foreign intruders (Marmaras and Lampropoulou, 2009); melanization and coagulation or clotting of the hemolymph (the insect analog of vertebrate blood), which require the active form of the enzyme phenoloxidase and the participation of humoral and cellular factors that lead to the rapid production and deposition of melanin around wounds, and foreign invaders (Eleftherianos and Revenis, 2011); generation of high levels of reactive oxygen species (ROS) and AMP in epithelial cells as well as nitric oxide (NO) that is also involved in the regulation of innate immune responses to bacteria and parasites (Ryu et al., 2010; Royet, 2011), which in some cases is stimulated by the gut microbiota; RNA interference (RNAi) and inducible innate immune responses against viral pathogens (Kemp and Imler, 2009). In addition, physiological and ecological factors that include dietary nutrition and energy metabolism, feeding behavior, circadian rhythms, aging, mating success and reproductive activity, lifestyle, and immune priming also affect the host immune response to pathogenic challenges (Sadd and Schmid-Hempel, 2009; Schneider, 2009; Chambers and Schneider, 2012).

Apart from the native microbiota, insects also carry symbiotic bacteria that occupy specific cells and tissues within the host. These symbiotic microbes live under the pressure of an active immune system and therefore they must devise strategies that allow them to withstand the adverse effects of host immune defense mechanisms (Gross et al., 2009; Douglas, 2011; Weiss and Aksoy, 2011). Furthermore, exposure of insects to microbes at all stages of their life cycle could have shaped the insect immune system to fight infection against pathogenic organisms (Mateos et al., 2006). Recent findings strongly suggest that the presence of symbiotic bacteria in various insect species is associated with increased host resistance to various pathogens and parasites. The results of these studies are of particular interest for developing alternative strategies to transgenic approaches for the efficient management of noxious insects.

## Endosymbiotic bacteria in insects

Almost all insects are associated with heritable endosymbiotic bacteria. Many endosymbionts are able to form a mutualistic relationship with their host and others can cause severe effects on various biological functions of their insect partner (Feldhaar and Gross, 2009). Primary endosymbionts are vertically transmitted from mother to offspring and they provide their hosts with specific nutritional compounds that are important for their survival and development. For example, *Buchnera aphidicola* endosymbiotic bacteria of the pea aphid, *Acyrthosiphon pisum*, synthesize essential amino acids that the aphids cannot receive from the plant sap. Similarly, *Wigglesworthia glossinidia* endosymbionts of tsetse flies (*Glossina morsitans*) produce essential vitamins that are not present in the vertebrate blood meal (Aksoy and Rio, 2005; Oliver et al., 2010). Insects have co-evolved with their primary endosymbionts for several million years; therefore their relationship is obligate. This means that insects lacking their bacteria are unable to grow and reproduce while the symbiotic bacteria are not viable in the absence of their host (Kikuchi, 2009). Primary endosymbionts are mainly found in bacteriocytes, which are specialized cells that provide nutrients to the bacteria and they are contained within the bacteriome (Bright and Bulgheresi, 2010). Secondary endosymbionts can be transmitted horizontally, vertically or via the environment. These are commensal bacteria that have evolved symbiotic relationships with their hosts more recently and can be found in the hemocoel (insect body cavity containing the hemolymph and organs) (Wernegreen, 2012). For example, *Sodalis glossinidius* bacteria are secondary symbionts in the tsetse fly, which are maternally transmitted to the progeny and can be found inter- and intra-cellularly in various tissues of the fly (Balmand et al., 2012). *Sodalis* was the first insect endosymbiont that was reported to be successfully isolated and cultured *in vitro* (Matthew et al., 2005).

The most commonly found facultative endosymbiotic bacteria are *Wolbachia* and *Spiroplasma*. *Wolbachia* is a group of maternally transmitted intracellular alpha-proteobacteria that infect a wide range of insects as well as filarial nematodes. These symbionts are able to manipulate the reproductive properties of their insect hosts by inducing parthenogenesis, male-killing, feminization and, most commonly, cytoplasmic incompatibility (Werren et al., 2008; Saridaki and Bourtzis, 2010). *Spiroplasma* symbionts are wall-less, motile, helical, gram-positive bacteria that associate both endocellularly and extracellularly with a variety of arthropods, particularly insects. Some species of *Spiroplasma* bacteria cause female-biased sex ratios of their host insects including *Drosophila* flies, ladybird beetles, and butterflies, as a result of selective death of the male offspring during embryogenesis (Regassa and Gasparich, 2006; Haselkorn, 2010). The consequence of the diversity of hosts and symbioses is that *Wolbachia* and *Spiroplasma* endosymbionts must evade a broad range of host immune defense mechanisms to ensure their survival and transmission, and the host must regulate the bacterial population to avoid the effects of pathogenicity or fitness costs. On the contrary, the absence of other heritable endosymbionts apart from *Wolbachia* and *Spiroplasma* in *Drosophila* perhaps highlights the ability of the fly immune system to control infections by other symbiotic bacteria (Mateos et al., 2006).

## Interaction of endosymbionts with the insect immune system

*Wolbachia* and *Spiroplasma* endosymbionts can be found in the insect hemolymph and thus they can interact directly with secreted molecules of the humoral immune response (Dobson et al., 1999; Haselkorn, 2010). A previous study examined the transcription of AMP genes in *Drosophila simulans* flies and *Aedes albopictus* mosquitoes carrying or lacking *Wolbachia* endosymbionts (Bourtzis et al., 2000). Results showed that *Diptericin* and *Cecropin* genes were not up-regulated in *Wolbachia* infected flies compared to uninfected controls. Similarly, *Wolbachia* infection failed to induce *Defensin* gene transcription in mosquitoes. These findings indicate that *Wolbachia* endosymbionts do not activate or repress AMP gene transcription in these insect species. Another work tested the transcriptional level of immune genes in *Drosophila* adults artificially infected with *Spiroplasma* (strain NSRO) and in uninfected control flies (Hurst et al., 2003). It was observed that the bacteria caused no up-regulation of AMP genes in infected flies, the presence of *Spiroplasma* did not down-regulate the immune response of the fly against heat-killed bacteria and fungal spores, and endosymbiotic bacterial load decreased in flies overexpressing *Toll* or flies receiving a septic challenge. These results suggest that *Spiroplasma* bacteria are undetected or they are efficiently restrained by the fly immune system. However, more recently it was reported that titers of the native *Spiroplasma* strain MSRO are not affected in *Drosophila* lines carrying null mutations that affect key components of the Toll and Imd immune pathways compared to wild type flies, whereas induction of the systemic immune response by microbial infection or ectopic activation leads to high titers of the endosymbionts (Herren and Lemaitre, 2011). Another study examined the effect of male-killing and non-male-killing *Spiroplasma* strains (NSRO and NSRO-A, respectively) on the *Drosophila* immune response (Anbutsu and Fukatsu, 2010). The authors found that neither *Spiroplasma* strain was able to induce up-regulation of AMP genes in unchallenged flies. Although in flies challenged with dead bacteria or a fungus there was no up-regulation or down-regulation of immune genes by either *Spiroplasma* strain, flies carrying the male-killing strain showed lower AMP gene transcription levels compared to uninfected controls. Furthermore the male-killing strain was able to proliferate in old *Toll* gain-of-function mutant flies. These results imply that the close association of *Spiroplasma* endosymbionts with *Drosophila* is based on specific strategies that the bacteria employ in order to evade, suppress, and tolerate the host immune response.

The interaction of endosymbiotic bacteria with insect immune recognition genes has previously been established by analyzing the response of the weevil *Sitophilus zeamais* to its intracellular symbiont, *Sitophilus zeamais* primary endosymbiont (SPE) (Anselme et al., 2006). It was shown that transcription of peptidoglycan recognition protein gene (*PGRP*) (ortholog of the *DrosophilaPGRP-LB*) was increased in the bacteriome and this increase coincides with the release of the endosymbiotic bacteria from the bacteriocytes. In turn, high *PGRP* gene transcription at the nymphal stage of *Sitophilus* is accompanied by significant up-regulation of the endosymbiont virulence genes (Dale et al., 2002). Interestingly, SPE injection into the hemolymph of *Sitophilus* triggers a systemic immune activation, whereas persistent SPE infection of bacteriocytes results in the transcriptional induction of the AMP gene *Coleoptericin A* (*ColA*) (Anselme et al., 2008). It was recently demonstrated that silencing of *ColA* by RNAi affects the amount of SPE that exits the bacteriome. This exciting finding implies that this AMP acts not only in response to foreign microorganisms but also regulates bacterial symbiosis in the weevil (Login et al., 2011).

Transcriptional analyses of the interaction between endosymbiotic bacteria and insect host genes have recently been performed *in vivo* and *in vitro*. A microarray study on gene transcription of *Drosophila melanogaster* larval testes has shown that several genes related to humoral and cellular host immune response were up-regulated in the presence of the naturally occurring avirulent *w*Mel strain of *Wolbachia* (Zheng et al., 2011), including two AMP genes (*Drosomycin* and *Lysozyme*) and a positive regulator in the immune deficiency (Imd) signaling pathway (*Kenny*). Similarly, transcription profiles of *Wolbachia* infected and uninfected *Drosophila* S2 cells revealed the up-regulation of several genes involved in the Imd, Toll, and c-Jun N-terminal kinase (JNK) pathways (Lemaitre and Hoffmann, 2007), such as the NF-κB transcription factors *Relish*, *Dorsal*, and *dJun* (Bohmann et al., 1994; Hetru and Hoffmann, 2009), the sole *Drosophila* JNK-specific MAPK phosphatase puckered (McEwen and Peifer, 2005), and various AMP genes (Xi et al., 2008). In sharp contrast, transcriptomic characterization of *Anopheles gambiae* cells transinfected with the *Wolbachia* strains *w*Ri and *w*AlbB showed down-regulation of over 75% of the immune related genes involved in pathogen recognition and signaling cascades, and genes encoding effector molecules (Hughes et al., 2011b). Another *in vitro* study used a silkworm microarray to investigate transcription of *Bombyx mori* cells infected by *Cardinium* endosymbiotic bacteria and the *Wolbachia* strain *w*Str, an endosymbiont of the small brown planthopper *Laodelphax striatellus* (Nakamura et al., 2011). Infection with *Cardinium*-induced the transcription of AMP genes, a serine protease gene that participates in the activation of the prophenoloxidase cascade, and two pattern recognition protein genes, while infection with *Wolbachia* did not activate any immune related genes. Crucially, a recent investigation tested the role of *Wolbachia* Surface Protein (WSP) in the interaction between the endosymbiont and immune genes in mosquitoes. The authors used cell lines from *A. gambiae*, which is not a natural host for *Wolbachia*, and *A. albopictus*, which is naturally infected with the strain *w*AlbB, and compared the transcriptional induction of immune genes between the two cell lines (Pinto et al., 2012). The *A. gambiae* cell-lines showed strong transcriptional up-regulation of certain AMP genes as well as complement-like genes that participate in the elimination of *Plasmodium* parasites in this mosquito species. In contrast, lower mRNA levels of immune genes were detected in cell-lines from *A. albopictus*. Interestingly, transcriptional levels in the latter case was dependent on WSP concentration and was mainly restricted to early time points post WSP treatment.

In the parasitoid *Asobara tabida*, regulation of immune genes by the native *Wolbachia* strains *w*Atab1, *w*Atab2, and *w*Atab3 was found to be tissue and sex-specific (Kremer et al., 2012). Numerous upstream genes in the Imd, Toll, JNK, RNAi, and Janus Kinase/Signal Transducer Activator of Transcription (JAK/STAT) pathways were significantly up-regulated in male wasps harboring *Wolbachia* compared to downstream AMP genes that were mostly down-regulated, while immune genes were transcribed at lower levels in the ovaries of females wasps. The authors concluded that *Wolbachia* endosymbionts can influence the immune response of the wasp to facilitate persistence and maintain close association with their host. A transcriptomic experiment in the pea aphid *A. pisum* to test the effect of the facultative symbiont *Serratia symbiotica* on immune gene transcription in infected and uninfected aphids reported that there were no significant changes in transcription of immune genes. These included recognition genes, AMP genes, thioester-containing protein (TEP) genes, prophenoloxidase, and NO cascade genes (Burke and Moran, 2011). These data support the notion that *Serratia* endosymbionts are not recognized by the pea aphid immune system. High variation in immune gene transcriptional regulation in the above studies leads us to speculate that endosymbiotic bacteria may adopt different strategies to actively evade the host immune system and promote infection.

It has further been proposed that endosymbiotic bacteria can also interact with the insect cellular immune response and melanization response. It was recently shown that pea aphid lines carrying different secondary symbionts contain variable number of adherent hemocytes (Schmitz et al., 2012). In particular, aphids carrying *Hamiltonella defensa* or *Regiella insecticola* symbiotic bacteria contained fewer numbers of hemocytes compared to aphids carrying *S. symbiotica* or no endosymbionts. Furthermore, *B. aphidicola* primary endosymbionts were found in phagolysosomes of adherent hemocytes. These data suggest that different endosymbionts can interact in different ways with host immune cells and the identification of endosymbionts in hemocytes might form a mechanism for their successful transmission from the parent aphids to the offspring. Of note, removal of hemocytes from *Drosophila* through genetic manipulations was not directly linked to variation in *Spiroplasma* titers in the hemolymph of the fly (Herren and Lemaitre, 2011), and the effect of endosymbionts on melanization levels were found to be higher in *Aedes aegypti* mosquitoes transinfected with the *Wolbachia* strain *w*MelPop compared to uninfected controls (Thomas et al., 2011). Interestingly, control mosquitoes laid darker eggs compared to those deposited by *Wolbachia* infected individuals, and there were similar levels of dopamine in mosquitoes carrying or lacking *Wolbachia* endosymbionts. Similar results were also observed in *D. melanogaster* and *D. simulans* flies naturally infected with the *Wolbachia* strains *w*Mel and *w*MelPop. These results further emphasize that endosymbiotic bacteria are able to regulate the intensity of key immune defense mechanisms, like melanization, in their insect hosts.

## Endosymbionts and the insect immune response to pathogens

### Effect of endosymbionts on bacterial infections

The impact of *Wolbachia* and *Spiroplasma* endosymbionts to insect antibacterial immune responses has recently drawn the attention of insect immunologists. A comprehensive study has lately investigated the involvement of *Spiroplasma* in the response against Gram-positive and Gram-negative bacterial pathogens (Herren and Lemaitre, 2011). This work revealed that flies carrying the *Spiroplasma* strain MSRO, which naturally infects *D. melanogaster*, were more susceptible to septic injury with the Gram-negative bacteria *Erwinia carotovora* and *Enterobacter cloacae*, but not with the Gram-positive bacterium *Enterococcus faecalis* or the fungus *Beauveria bassiana*, compared to flies lacking the endosymbiont. These results strongly suggest that *Spiroplasma* endosymbionts are potentially able to alter the sensitivity of flies to some bacterial pathogens. Furthermore, there was no difference in Toll or Imd pathway activation between flies containing or lacking *Spiroplasma* upon infection with the bacteria *Micrococcus luteus* (Gram-positive) or *E. carotovora* (Gram-negative), respectively. Surprisingly, infection of wild type flies with the plant pathogenic bacterium *Spiroplasma citri* failed to activate the immune system, leading to bacterial proliferation in the hemolymph and death of the flies. In addition, wild type and immune mutants infected by *S. citri* showed no differences in their survival rates following infection with this bacterium. The ability of *S. citri* to kill flies is probably due to the fact that these bacteria are not detected by the *Drosophila* immune system.

**Figure 1:**
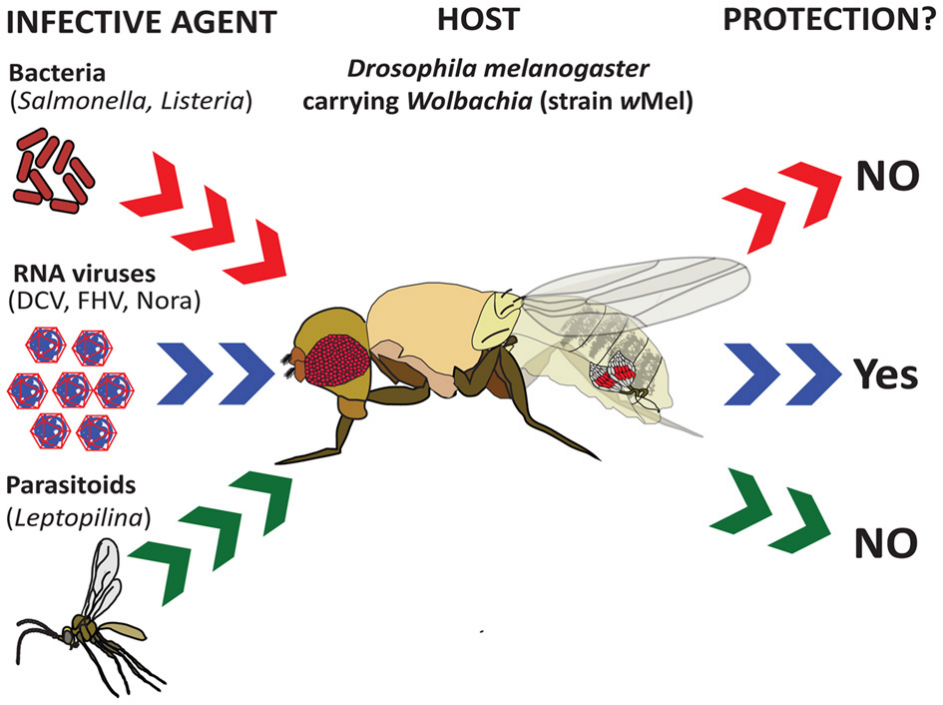
**Effect of *Wolbachia* endosymbiotic bacteria on the ability of *Drosophila* to resist infection.** Recent studies have shown that the presence of *Wolbachia* strain *w*Mel in *Drosophila melanogaster* confers resistance to infection by various RNA viruses (*Drosophila* C Virus, Flock House Virus, and Nora virus) (Teixeira et al., 2008), but not by intracellular bacterial pathogens (*Salmonella typhimurium* and *Listeria monocytogenes*) (Rottschaefer and Lazzaro, 2012) or parasitoid wasps (*Leptopilina boulardi*) (Martinez et al., 2012).

**Figure 2:**
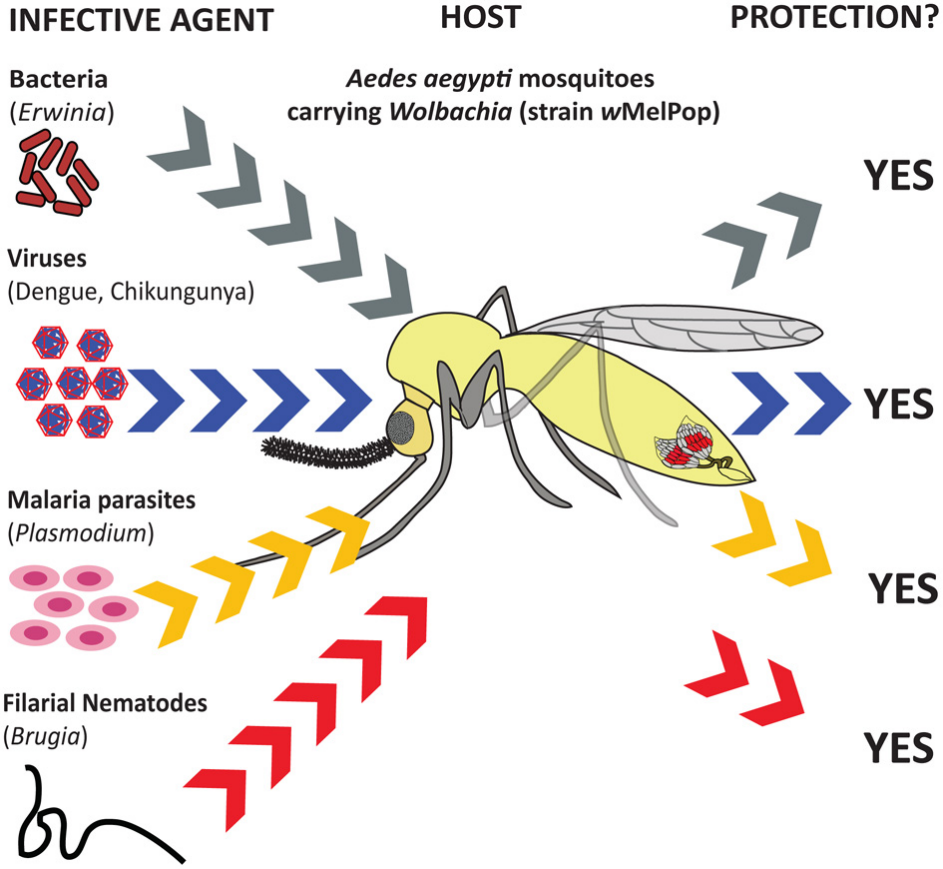
**Influence of *Wolbachia* endosymbionts on inhibition/reduction in transmission capacity as well as protection of mosquitoes against infection.** *Aedes aegypti* mosquitoes transinfected with the *Wolbachia* strain *w*MelPop are protected from infection by pathogenic bacteria (*Erwinia carotovora*) (Kambris et al., 2009), viruses (dengue and Chikungunya) (Moreira et al., 2009), malaria parasites (*Plasmodium gallinaceum*) (Moreira et al., 2009), and parasitic filarial nematodes (*Brugia pahangi*) (Kambris et al., 2009).

Challenge of *Wolbachia*-infected *D. melanogaster* and *D. simulans* flies (strains *w*Au, *w*Ri, *w*No, *w*Ha, and *w*MelCS, respectively), with three gram-negative bacterial pathogens (*Pseudomonas aeruginosa* PA01, *Serratia marcescens*, and *E. carotovora*) did not affect their survival ability compared to *Wolbachia*-free control flies (Wong et al., 2011). In addition, no differences in pathogen load and transcription of AMP genes was found between the flies carrying or lacking *Wolbachia* endosymbionts. These results suggest that native *Wolbachia* endosymbionts do not confer antibacterial immune priming in *Drosophila*. Apart from studies testing the effect of *Wolbachia* on survival of flies infected with extracellular bacteria, similar research has lately been carried out using intracellular bacterial pathogens. Experiments involving infection of *D. melanogaster* flies naturally infected with *Wolbachia* (probably strain *w*Mel) with two intracellular bacterial pathogens, *Salmonella typhimurium* and *Listeria monocytogenes*, and an extracellular pathogen, *Providencia rettgeri*, showed no differences in pathogen load between the two types of flies (Rottschaefer and Lazzaro, 2012) (Figure 1). These results indicate that the presence of *Wolbachia* in *Drosophila* does not affect replication of these intracellular pathogens in the fly. Similar studies have also expanded to important insect vectors of human diseases. In particular, *A. aegypti* mosquitoes transinfected with the *Wolbachia* strain *w*MelPop showed increased resistance to infection with *E. carotovora* bacteria (Kambris et al., 2009) (Figure 2). This effect was probably due to up-regulation of immune effectors, such as AMP molecules, in the *Wolbachia*-infected mosquitoes. Although these results imply that *Wolbachia* can potentially protect against bacterial infections, additional tests with other bacterial pathogens and knockdown studies would be extremely useful for verifying this hypothesis. Furthermore, the results in *Drosophila* and mosquitoes indicate that native and experimentally introduced *Wolbachia* endosymbionts modulate immune responses in distinct manners in different hosts.

The immune response of tsetse flies to infection with symbiotic and foreign bacteria was previously examined (Weiss et al., 2008). The authors infected tsetse flies with *E. coli* and *S. glossinidius* bacteria and tested the transcriptional levels of several immune related genes in the host. They found that infection with virulent *E. coli* K12 bacteria failed to induce transcriptional activation of immune genes whereas infection with non-virulent wild type *S. glossinidius* bacteria resulted in up-regulation of several immune genes. It was also shown that *E. coli* was cleared from the hemolymph, probably because these bacteria are unable to manipulate host immune responses to their advantage, and *S. glossinidius* persisted, probably due to increased resistance of the bacteria to host immune effector molecules. The same research group recently performed elegant experiments using tsetse flies lacking their endogenous symbiotic microbes (aposymbiotic flies) and showed that they were sensitive to systemic infection with *E. coli* bacteria (Weiss et al., 2012). This sensitivity was attributed to down-regulation of numerous immune genes and in particular those associated with the cellular immune response as well as to the absence of phagocytic hemocytes. However, transfer of hemocytes from wild type to aposymbiotic flies prior to challenge with *E. coli* reversed the susceptible phenotype. The resistant phenotype was also observed when aposymbiotic offspring of symbiont-cured female flies were fed on diet supplemented with cell extracts from tsetse's obligate symbiont, *Wigglesworthia*. These findings suggest that *Wigglesworthia* bacteria participate actively in the development and activation of the immune system of their host.

Transcriptional analysis of immune genes that are regulated in *Sitophilus* larvae carrying or lacking SPE endosymbiotic bacteria upon infection with *E. coli* bacteria provided further insight into the role of endosymbionts in regulating the host immune response (Vigneron et al., 2012). Surprisingly, it was reported that larvae without the endosymbionts exhibited stronger induction of several immune related genes, including AMP genes and canonical components of immune signaling pathways, compared to symbiotic insects. This is probably due to manipulation of host immune gene expression by the endosymbiont or due to differential immune activity by the host in order to maintain the endosymbionts in the bacteriome and trigger a response to foreign microbes.

### Effect of endosymbiotic bacteria on viral infections

There has been a dramatic increase in the number of publications on the interaction between endosymbiotic bacteria and the insect immune response to viral pathogens. It was originally shown that *D. melanogaster* wild type adult flies naturally infected with the *Wolbachia* strains *w*MelCS and *w*MelPop survive longer an infection by the RNA viruses *Drosophila* C Virus (DCV), Flock House Virus (FHV) and Cricket Paralysis Virus (CrPV) (Hedges et al., 2008). A concurrent study verified and extended these results by reporting increased resistance of *Wolbachiaw*Mel infected *D. melanogaster* flies to Nora virus (RNA virus), but not to Insect Virus 6 (DNA virus), and reduced viral burden in flies carrying the endosymbionts (Teixeira et al., 2008) (Figure 1). The *Wolbachia*-mediated protection against DCV and FHV is independent of the small interfering RNA pathway that inhibits viral replication by sequence-specific degradation of viral RNA (Ghildiyal and Zamore, 2009). In particular, it was shown that *Dicer-2*, *Argonaute2*, and *r2d2* loss-of-function mutant flies naturally carrying *Wolbachiaw*Mel endosymbionts succumbed at a far slower rate to infection by DCV and FHV compared to mutants free of the endosymbionts (Hedges et al., 2012).

The antiviral effect of *Wolbachia* is not restricted to *D. melanogaster* flies only. It has been shown that *D. simulans* flies naturally harboring *Wolbachia* endosymbionts can also be protected against DCV and FHV infection, but the protection varies with different strains of *Wolbachia* (Osborne et al., 2009). The three *Wolbachia* strains that gave strong avtiviral protection in *D. simulans* were *w*Mel, *w*Ri and *w*Au, but not the strains *w*Ha and *w*No. However, increased protection to DCV does not necessarily reflect lower levels of viral load in the *Wolbachia* infected flies. The authors further found increased density of the three *Wolbachia* strains that confer antiviral protection to *D. simulans*. Interestingly, a male-killing native *Wolbachia* strain does not protect *D. bifasciata* flies from DCV and FHV (Longdon et al., 2012). This probably indicates genetic differences between the male-killing *Wolbachia* strain and those that offer antiviral protection to their hosts. Recent data also demonstrate that the presence of *w*Mel related *Wolbachia* in *D. melanogaster* is beneficial because the endosymbiont is able to inhibit replication of bluetongue virus, which otherwise replicates efficiently in flies free of *Wolbachia* (Shaw et al., 2012). However, the genetic basis of this effect is currently unclear.

*Wolbachia* endosymbionts can also have detrimental effects to their hosts. Recent laboratory and field investigations suggest that the *Wolbachia* strain *wExe1*, *wExe2*, and *wExe3* naturally found in larvae of the African armyworm, *Spodoptera exempta*, significantly enhances the mortality caused by a double-stranded DNA baculovirus (Graham et al., 2012). The authors speculate that this could be due to the eco-evolutionary dynamics of the interaction between *Wolbachia* and the baculovirus, which are both obligate intracellular symbionts in the armyworm but they use distinct modes of transmission.

Several studies have also examined the interaction between *Wolbachia* infection in mosquitoes and their vector competence for important mammalian viral pathogens. It was originally found that introduction of the *w*MelPop-CLA *Wolbachia* strain into *A. aegypti* reduces the ability of two arboviruses (dengue virus and Chikungunya virus) and the avian malaria parasite (*Plasmodium gallinaceum*) to establish infection in the mosquito (Moreira et al., 2009) (Figure 2). The resistant phenotype was attributed to the approximately 100-fold up-regulation of genes encoding immune effectors, such as the AMP Cecropin and Defensin, TEP proteins, and C-type lectins, in *Wolbachia* infected mosquitoes compared to uninfected controls or competition for important host cell components. Of note, transinfection of old *A. aegypti* mosquitoes with the *w*MelPop strain of *Wolbachia* results in changes in virulence that lead to behavioral effects that in turn reduce blood-feeding efficiency (Turley et al., 2009). *A. aegypti* mosquitoes transinfected with *Wolbachia* were also found to suppress dengue virus replication, dissemination, and transmission compared to uninfected controls (Bian et al., 2010). These effects were associated with higher transcription of *Cecropin* and *Defensin*, as well as up-regulation of the Toll pathway genes *Rel1*, *Spz1A*, and *GNBP1* in *Wolbachia* infected mosquitoes, but not in those lacking the endosymbionts. *Wolbachia* infection in *A. aegypti* causes oxidative stress and high levels of ROS. It was recently shown that Toll pathway activation in *A. aegypti* transinfected with the *Wolbachia* strain *w*AlbB is also linked to the up-regulation of genes that control reduction-oxidation (redox) reactions (Pan et al., 2012). Transcriptional activation of redox related genes are important for preventing adverse effects of oxidative stress in the mosquito. These results imply that *Wolbachia* is capable of regulating the Toll pathway in *A. aegypti*; this might constitute a mechanism for controlling dengue infection in mosquitoes.

Certain *Wolbachia* strains (*w*Mel and *w*MelPop-CLA) in *D. melanogaster* have been introduced into *A. aegypti* mosquitoes where they have been found to increase the transcriptional levels of melanization genes as well as AMP and Toll related genes (Rancès et al., 2012). Up-regulation of immune genes in *Wolbachia* transinfected mosquitoes probably leads to over-replication of the bacteria in the new host that could result in immune priming. However, it was observed that although dengue accumulation is reduced in both *D. melanogaster* and *A. aegypti* infected with *Wolbachia*, there is no up-regulation of the same genes in the fly. These findings denote that transcriptional induction of the specific immune genes is probably not required to reduce dengue accumulation in the mosquito host. The lack of up-regulation of these immune genes by native *Wolbachia* endosymbionts in the fly with simultaneous suppression of dengue also suggests that reduction of virus accumulation in *Drosophila* is likely due to host physiological or metabolic responses. Such responses could interfere with pathogen replication and therefore alter the outcome of infection. Given that *w*Mel and *w*MelPop differentially regulate the expression of immune genes in the fly, it is further possible that distinct physiological/metabolic changes are caused by each *Wolbachia* strain. Another possibility could be that native *Wolbachia* strains in *Drosophila* have different needs for nutritional supplies or competition for resources between the endosymbionts and the pathogen could also modify the infection status and efficiency of the host immune response. These various possibilities need to be explored in future studies.

Inhibition of dengue replication in *A. aegypti* mosquitoes has also been confirmed *in vitro* using *A. aegypti* cell lines transinfected with the *w*MelPop-CLA *Wolbachia* strain (Frentiu et al., 2010). These experiments showed that mosquito cells proliferate at slower rates in the presence of *Wolbachia* and viral protection correlates with the density of the endosymbiotic bacteria contained in each cell. Interestingly, the *Wolbachia*-mediated protective effect against dengue was not observed in *Aedes albopictus* mosquitoes that naturally carry the *w*AlbA and *w*AlbB strains of the endosymbiont (Lu et al., 2012). Using *in vivo* and *in vitro* assays the authors showed that lack of *A. albopictus* resistance to dengue virus depends strongly on *Wolbachia* density that is extremely low in midgut, fat body and salivary glands compared to *A. aegypti*. Another study also recently reported that although *A. albopictus* transmits dengue in the presence of its naturally occurring *Wolbachia* endosymbionts, transmission of the virus is blocked in *w*Mel transinfected mosquitoes in the absence of significant immune gene up-regulation (Blagrove et al., 2012). These results suggest that dengue transmission inhibition by *A. albopictus* appears to be limited to lines harboring specific exogenous strains of *Wolbachia*. In the same mosquito species, replication of Chikungunya virus was increased after 4 days of infection with simultaneous decrease in density of naturally occurring *Wolbachia* endosymbionts (strains *w*AlbA and *w*AlbB) (Mousson et al., 2010). This phenotype was observed in whole mosquitoes as well as in the midgut and salivary gland tissues that play a key role in viral transmission. Strikingly, there was high variation in viral load in *A. albopictus* free of *Wolbachia*, which indicates that the endosymbiont is not probably required for Chikungunya replication in this mosquito. A similar recent study in *A. albopictus* has also confirmed that *Wolbachia* numbers decrease with infection by Chikungunya (Zouache et al., 2012). In another mosquito vector, the southern house mosquito *Culex quinquefasciatus*, natural *Wolbachia* infection improves resistance to West Nile virus by lowering viral titers and transmission during feeding compared to mosquitoes without *Wolbachia* (Glaser and Meola, 2010). Interestingly, the authors reported that the protective effect conferred by *Wolbachia* was more pronounced in *Drosophila* flies infected with the virus compared to *C. quinquefasciatus* mosquitoes, but the molecular basis of this difference was not investigated.

### Effect of endosymbiotic bacteria on parasitic infections

Recent efforts to examine the potential role of endosymbiotic bacteria in the insect immune response to parasites have mainly focused on *Wolbachia* and *Wigglesworthia*. A study involving *Wolbachia* infected *A. gambiae* cell-lines and intrathoracic inoculation of endosymbionts into adult mosquitoes showed that the presence of the endosymbiont increased the transcription of selected immune genes (e.g., *TEP1*) compared to control treatments (Kambris et al., 2010). Gene up-regulation resulted in lower number of *Plasmodium berghei* oocysts in the *Wolbachia*-infected mosquitoes. Furthermore, silencing of *TEP1* in mosquitoes containing the endosymbiont increased oocyst numbers, which demonstrates that *Wolbachia*-induced over-transcription is an important factor for the inhibition of the parasite. Similar experiments also showed reduced numbers of *Brugia pahangi* parasites in *A. aegypti* somatically infected with *Wolbachia*.

It is of particular interest to note that not all *Wolbachia* strains confer pathogen resistance to their hosts. It was recently observed that the *Wolbachia* strain *w*AlbB from *A. albopictus* mosquitoes does not affect the survival of somatically infected *A. gambiae* mosquitoes after feeding on *P. berghei* infected mice, whereas the strain *w*MelPop from *D. melanogaster* reduces the survival of mosquitoes after blood feeding (Hughes et al., 2012). In addition, strain *w*AlbB increases the numbers of *P. berghei* oocysts in the *A. gambiae* midgut but strain *w*MelPop decreases oocyst numbers, while *w*MelPop reaches higher densities than *w*AlbB in infected mosquitoes. The authors conclude that these phenotypic differences in the anti-*Plasmodium* response of *A. gambiae* carrying different strains of *Wolbachia* may reflect the effect of the endosymbiont on the mosquito immune response. In a similar study, *w*MelPop strain was found in several tissues throughout the mosquito, but it was not detected in the gut and ovaries (Hughes et al., 2011a). It was further shown that *w*MelPop and *w*AlbB strains can substantially reduce *Plasmodium falciparum* oocysts in the *A. gambiae* gut, *Wolbachia* infection differentially regulates several immune genes in the mosquito, and that both strains efficiently replicate in their host. The authors propose that these findings could be useful for employing *Wolbachia* as a means to reduce transmission of *Plasmodium* parasites. In agreement with previous results, two strains of *Wolbachia pipientis*, *w*Pip(Sl) and *w*Pip(Mc), naturally found in *Culex pipiens* were also shown to confer protection to mosquitoes following infection with *Plasmodium relictum* parasites (Zélé et al., 2012).

*Wolbachia* endosymbionts in mosquitoes can also affect the development of other important human parasites, such as filarial nematodes. In particular, whole-genome microarray experiments demonstrated that transinfection of *A. aegypti* with the *w*MelPop strain of *Wolbachia* leads to up-regulation of a considerable number of genes (especially *Cecropins* and other AMP or effector genes) compared to uninfected mosquitoes, which may be responsible for the inhibitory effect on the development of *B. pahangi* parasites (Kambris et al., 2009) (Figure 2).

In *Drosophila*, it was recently nicely demonstrated that mushroom-feeding *D. neotestacea* flies harboring *Spiroplasma* endosymbionts show increased tolerance to its natural nematode parasite *Howardula aoronymphium* in both the wild and the lab (Jaenike et al., 2010). The authors showed that the presence of *Spiroplasma* can rescue the fertility of female *D. neotestacea* flies parasitized with the nematodes. This rescue is not observed in *Wolbachia*-infected or endosymbiont-free flies parasitized with the nematodes. This tolerant phenotype was attributed to an unknown mechanism that reduces the growth and reproductive ability of the worms in the *Spiroplasma*-infected flies. It was further shown that numbers of *Howardula* nematodes decreases in *D. neotestacea* populations carrying *Spiroplasma*, but spread in populations lacking the endosymbiont (Jaenike and Brekke, 2010). These results imply that *Spiroplasma* endosymbionts have a major influence on the population dynamics of the host and its natural parasite.

In tsetse flies, the absence of native *Wigglesworthia* endosymbionts was originally shown to increase the numbers of trypanosome parasites and therefore significantly affected the vectorial competence of the flies (Pais et al., 2008). Protection of tsetse flies to trypanosome parasites has been attributed to the level of *PGRP-LB* gene transcription (Wang et al., 2009). *Wigglesworthia* induces the transcription of *PGRP-LB* in the bacteriome of the tsetse fly compared to flies lacking the endosymbiont. Interestingly, challenge of wild type or *relish*-silenced flies with *E. coli* bacteria does not affect *PGRP-LB* mRNA levels in the bacteriome, which suggests that transcription of *PGRP-LB* in the bacteriome is regulated by the endosymbiont. However, the molecular mechanism responsible for this effect is currently unknown. Transcription of *PGRP-LB* also affects symbiotic homeostasis since density of *Wigglesworthia* endosymbionts decreases when *PGRP-LB* is silenced. Furthermore, *PGRP-LB* transcription is important for resistance to trypanosome infection and transmission of the parasite. A more recent work has shown that *PGRP-LB* prevents immune activation and is therefore a crucial factor for protecting *Wigglesworthia* from detrimental effects on the host (Wang and Aksoy, 2012). In addition, tsetse larvae from *RGRP-LB*-silenced mothers produce adults with reduced immune capacity. Finally, the authors nicely demonstrate that expression of recombinant PGRP-LB exhibit bactericidal and trypanocidal activity that potentially mediate the immune response of tsetse to infections.

### Effect of endosymbiotic bacteria on parasitoid attacks

The effect of *Wolbachia* and *Spiroplasma* endosymbionts on the immune response of *Drosophila* against parasitoids has also been a subject for investigation. In a previous study, the encapsulation ability of *D. simulans* larvae naturally infected with *Wolbachia* was estimated upon infection with the parasitoid wasp *Leptopilina heterotoma* (Fytrou et al., 2006). It was shown that larvae carrying *Wolbachia* were less able to encapsulate parasitoid eggs compared to larvae lacking the endosymbiont, which suggests that the presence of *Wolbachia* in *Drosophila* suppresses the host cellular immune response against parasitism. Crucially, the mechanistic basis of this effect was not investigated. In addition, the authors observed that the presence of *Wolbachia* has no effect on the survival of male adult flies infected by the entomopathogenic fungus *B. bassiana*. However, a similar recent work concluded that native *Wolbachia* strains in *D. melanogaster* (*w*Mel and *w*MelPop) and *D. simulans* (*w*Ri) larvae have no influence on the encapsulation of parasitoid eggs, although there is a minor decrease in parasitoid development in flies infected by the endosymbiont (Martinez et al., 2012) (Figure 1). In contrast to the previous results, *Spiroplasma* endosymbionts increase the ability of *Drosophila hydei* to survive infection by *Leptopilina* wasps (Xie et al., 2010). This protective effect was later attributed to the high reproductive capacity of *Spiroplasma*-infected flies under conditions of parasitoid challenge (Xie et al., 2011). Finally, it was recently shown that *Wolbachia* endosymbionts (strain ST 306) in the parasitoid wasp *Leptopilina victoria* do not affect encapsulation of its eggs by various *Drosophila* host species (Gueguen et al., 2012).

## Conclusions and future prospects

Despite impressive advances in the broad field of insect innate immunity, our understanding of the role of endosymbiotic bacteria in the host immune response to pathogenic infections remains incomplete. Previous and recent studies have started to determine the phenotypic response of various insects carrying endosymbionts to infection by bacterial and viral pathogens as well as parasites. These studies have substantially improved our understanding of the complex interactions between insects, their endosymbiotic bacteria and pathogenic organisms in the infection and host immunity processes. It will further be of particular interest to test the immune response of insects with or without endosymbionts to infection by entomopathogenic fungi, as there is currently clear conflict within the literature on the effect of *Wolbachia* on fungal infections (Fytrou et al., 2006; Panteleev et al., 2007). Another major challenge is to gain a substantially more detailed and comprehensive grasp on how exactly endosymbiotic bacteria regulate insect immune defense mechanisms against pathogens and parasites. The identity of these mechanisms can be vigorously investigated via genetic, molecular and genome-wide transcriptome analyses in various insect models. Such studies will lay the foundation for exploring whether endosymbiotic bacteria also cause functional changes in the immune system of vertebrate animals. An additional challenge is to characterize the interplay between different endosymbionts, such as *Wolbachia* and *Spiroplasma*, co-existing in an insect host and efficiency of the immune function. It will also be important to elucidate the precise mechanisms employed by endosymbiotic bacteria to modulate immune signaling in insects. Similar research will undoutedly reveal the relative contribution of endosymbiotic bacteria to the overall host immune response against various classes of pathogenic organisms.

From the practical point of view, the recent discovery that the presence of *Wolbachia* endosymbionts in mosquitoes has a direct effect on insect sensitivity to pathogenic infections has attracted the attention of scientists for the development of novel approaches for the control of human diseases (Hancock et al., 2011). For example, it was recently demostrated that *Wolbachia* introduced into *A. aegypti* resulted in successful invasion of natural populations of mosquitoes (Hoffmann et al., 2011; Walker et al., 2011). Such approaches can be potentially implemented in field practices for the effective disruption of dengue transmission by mosquitoes. Finally, a better understanding of insect-symbiont-pathogen interactions will lead to more efficient management strategies, particularly those involving integrated and biological control tactics, those seeking to reduce reliance on broad-spectrum insecticides, and those involving the better deployment of insect-specific pathogens.

### Conflict of interest statement

The authors declare that the research was conducted in the absence of any commercial or financial relationships that could be construed as a potential conflict of interest.
